# MOMMY study profile: An integrative early‐life multi‐omics cohort in China

**DOI:** 10.1002/imo2.70068

**Published:** 2025-12-05

**Authors:** Lin Zhang, Yingzhi Liu, Shilan Wang, Jessica Yuet‐Ling Ching, Wing Hung Tam, Ting Fan Leung, Tak Yeung Leung, Paul K. S. Chan, Joyce W. Y. Mak, Chun Pan Cheung, Hein Min Tun, Eugene B. Chang, Orlando DeLeon, Qitao Huang, Xiaoqian Chen, Huiyi Huo, Yinglei Miao, Pui Kuan Cheong, Ka Long Ip, Yuk Ling Yeung, Mei Kam Chang, Chunmei Lyu, Hongju Yang, Bona Li, Yushuo Fan, Yang Sun, Suhua Jiang, Siew Chien Ng, Francis Ka Leung Chan

**Affiliations:** ^1^ Microbiota I‐Center (MagIC) Hong Kong SAR China; ^2^ Department of Anaesthesia and Intensive Care, Faculty of Medicine The Chinese University of Hong Kong Hong Kong SAR China; ^3^ Li Ka Shing Institute of Health Sciences, State Key Laboratory of Digestive Disease, Institute of Digestive Disease The Chinese University of Hong Kong Hong Kong SAR China; ^4^ Department of Medicine and Therapeutics, Faculty of Medicine The Chinese University of Hong Kong Hong Kong SAR China; ^5^ Department of Obstetrics and Gynaecologist, Faculty of Medicine The Chinese University of Hong Kong Hong Kong SAR China; ^6^ Department of Paediatrics, Faculty of Medicine The Chinese University of Hong Kong Hong Kong SAR China; ^7^ Hong Kong Hub of Paediatric Excellence The Chinese University of Hong Kong Hong Kong SAR China; ^8^ The Chinese University of Hong Kong ‐ Baylor College of Medicine Joint Medical Centre Hong Kong SAR China; ^9^ Department of Microbiology, Faculty of Medicine The Chinese University of Hong Kong Hong Kong SAR China; ^10^ JC School of Public Health and Primary Care The Chinese University of Hong Kong Hong Kong SAR China; ^11^ Department of Medicine, Section of Gastroenterology, Hepatology, and Nutrition University of Chicago Chicago Illinois USA; ^12^ Department of Obstetrics and Gynaecologist The First People's Hospital of Foshan, Foshan Guangdong China; ^13^ Department of Paediatrics The First People's Hospital of Foshan Foshan China; ^14^ Division of Gastroenterology The First Affiliated Hospital of Kunming Medical University Kunming Yunnan China; ^15^ Yunnan Province Clinical Research Center for Digestive Diseases Kunming Yunnan China; ^16^ Shunde Hospital of Southern Medical University, Foshan Guangdong China; ^17^ Yunnan Geriatric Medical Center, Department of Geriatrics The First Affiliated Hospital of Kunming Medical University Kunming Yunnan China; ^18^ New Cornerstone Science Laboratory The Chinese University of Hong Kong Hong Kong SAR China; ^19^ Centre for Gut Microbiota Research The Chinese University of Hong Kong Hong Kong SAR China; ^20^ The D.H. Chen Foundation Hub of Advanced Technology for Child Health (HATCH), The Chinese University of Hong Kong Hong Kong SAR China

**Keywords:** early life, longitudinal cohort, microbiome, MOMMY

## Abstract

Large‐scale, prospective birth cohorts capturing the complex interplay between the gut microbiome, host biology, and environmental exposures are crucial to understanding early‐life health but remain scarce, particularly within Asian populations. To address this gap, we established the MOMMY cohort (The MOther‐infant Microbiota transmission and its link to long terM health of babY), a large, prospective birth cohort uniquely designed to investigate maternal‐paternal‐infant microbiota transmission and its impact on child health within the understudied Chinese population. MOMMY aims to recruit 20,000 families from three geographically and economically diverse regions across China. This cohort prospectively follows pregnant mothers, fathers, and their infants, with children up to 7 years of age. Since September 2019, we have systematically collected a comprehensive repository of longitudinal biospecimens—including maternal and infant stool, breast milk, cord blood, and parental blood—stored in an accredited biobank. This is complemented by extensive data on environmental exposures, diet, and health outcomes gathered through validated questionnaires and physician assessments. The MOMMY cohort's unique value lies in its unprecedented scale, geographic diversity, and its integrative multi‐omics design, which will combine metagenomic, metabolomic, immunologic, and epigenetic data. By creating this unique resource, MOMMY will elucidate how early‐life microbial and molecular trajectories, shaped by genetic and environmental factors, influence child development and disease risk, thereby filling a critical gap in global microbiome research.

## Highlights

- MOMMY cohort (The MOther‐infant Microbiota transmission and its link to long terM health of babY) is a large, prospective birth cohort.
- Since September 2019, we have systematically collected a comprehensive repository of longitudinal biospecimens and extensive environmental data.
- MOMMY will elucidate how early‐life microbial and molecular trajectories, shaped by genetic and environmental factors, influence child development and disease risk, thereby filling a critical gap in global microbiome research.

## Graphical abstract

MOMMY cohort (The MOther‐infant Microbiota transmission and its link to long terM health of babY) is a large, prospective birth cohort uniquely designed to investigate maternal‐paternal‐infant microbiota transmission and its impact on child health within the understudied Chinese population. MOMMY aims to recruit 20,000 families from three geographically and economically diverse regions across China. This cohort prospectively follows pregnant mothers, fathers, and their infants, with children up to 7 years of age.

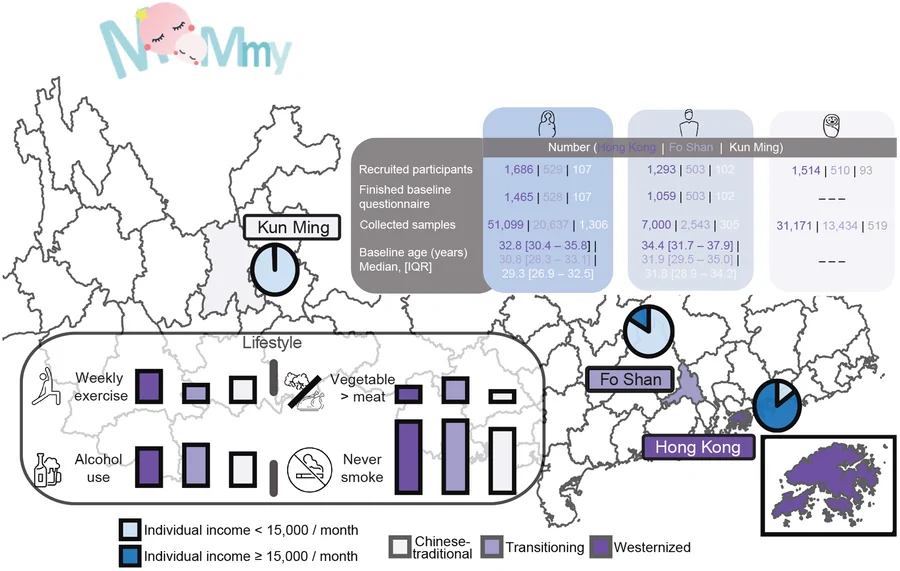

## 1 INTRODUCTION

Microbial colonization during infancy plays an instrumental role in healthy development [1, 2]. Emerging studies have reported an association between neonatal gut microbiome composition and the development of disease in children. These include autism spectrum disorders (ASDs), type 1 diabetes, asthma, and allergies [3, 4, 5, 6, 7, 8, 9, 10]. Due to the importance of the first 1000 days of life gut microbiota on healthy development, information garnered from birth cohort studies potentiates major public‐health and clinical impacts, including the management and treatment of these populations. Therefore, it is crucial to establish a biorepository containing samples before and during the onset of disease, combining a well‐curated cohort with robust longitudinal assessments of the microbiome that incorporate large population sizes and an inclusive diversity of participants.

Current understanding of the early life microbiome is largely skewed by Western cohorts, which are demographically distinct from Chinese populations [3, 6, 7, 10, 11, 12]. Owing to lifestyle, diet, and geographic factors, translation of those findings to Asia populations may be imprecise and hamper the generalizability of findings. However, there are fewer reports on the impact of early‐life microbial colonization on long‐term healthy development in large longitudinal follow‐up cohorts [13, 14]. Establishing a representative early life microbiome cohort in a family setting with longitudinal follow‐up in different urbanization levels is of great significance. Therefore, we launched a large‐scale, prospective birth cohort, MOther‐infant Microbiota transmission and its link to long terM health of babY (MOMMY, https://www.mommy.hk).

## 2 MOMMY COHORT ESTABLISHMENT

**Figure 1:**
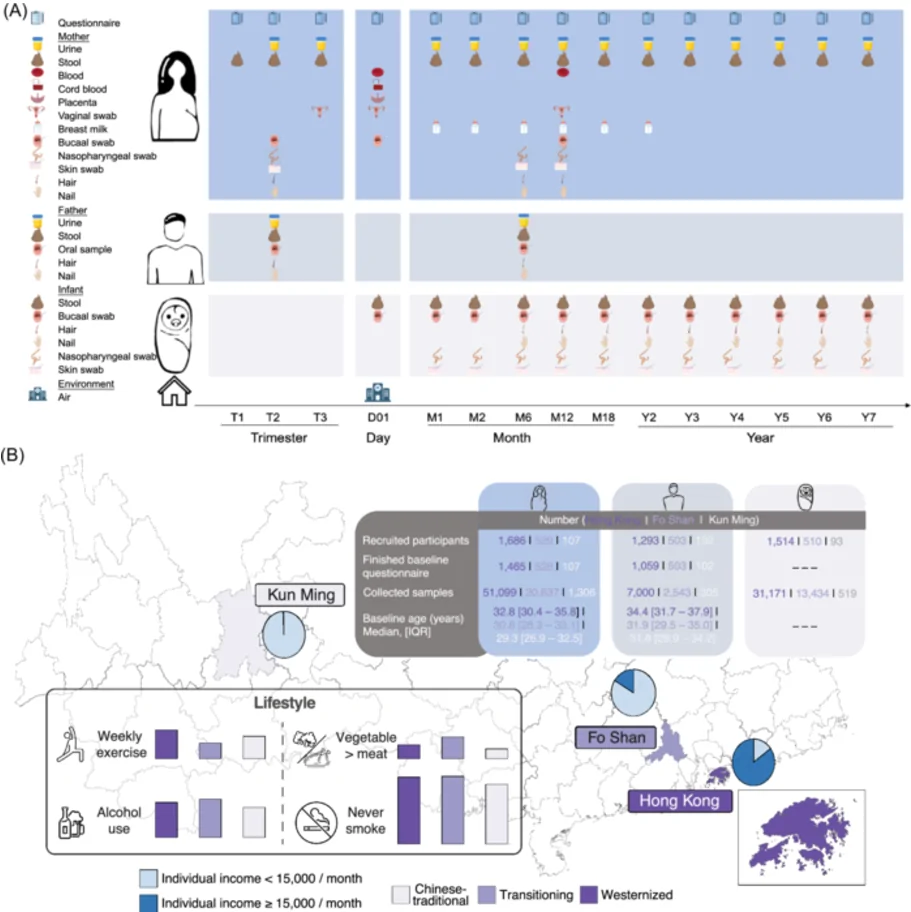
Overview of the MOMMY cohort. (A) The families enrolled are followed prospectively from pregnancy, delivery, and postnatal months 1, 2, 6, 12, 18, and years 2 to 7. (B) The demographics of subjects in the 3 MOMMY study sites.

MOMMY is a longitudinal, prospective birth cohort from the general population in China, which aims to recruit 20,000 pregnant mothers together with their partners and children with intensive follow‐up until the child reaches 7 years of age. The recruitment of pregnant women at trimesters 1, 2, or 3 started since September 2019. This study has been registered at ClinicalTrials.gov (NCT04117321) and has been approved by the Joint Chinese University of Hong Kong – New Territories East Cluster Clinical Research Committee (reference number 2019.243). Together with the Department of Obstetrics and Gynecology and the Department of Paediatrics at the Chinese University of Hong Kong, we have established standard operating procedures (SOP) to recruit pregnant mothers, their spouses and infants, and collect associated clinical metadata and biosamples as well as assess health outcomes (Tables 1, 2). The families enrolled are followed prospectively from pregnancy, delivery, and postnatal months 1, 2, 6, 12, 18, and year 2 to 7 (Figure 1A). The feasibility of this large‐scale collection is supported by two previous birth cohort studies, the Stool Microbiome and Allergic ReacTion (SMART Baby) and Intestinal Microbiota on Allergy, Growth and Development of the Next Generation in Hong Kong (SMART Gen HK) [15, 16, 17].

**Table 1:** Overview of bio‐samples collected in the MOMMY study.

Biosamples	Sample type	Pregnancy (trimester)			Birth/before discharge	Neonatal (months)		Infancy (months)				Childhood (years)
		1	2	3		1	2–3	6	12	18	24	3–7
Mother												
Urine	N/A	√	√	√		√	√	√	√	√	√	√
Stool	Fresh/Norgen preservative	√	√	√		√	√	√	√	√	√	√
Blood	Buffy coat				√				√			
	Serum				√				√			
	Plasma (EDTA)				√							
Cord blood	Cord buffy coat				√							
	Cord serum				√							
	Cord plasma (EDTA)				√							
Placenta	Placenta				√							
	Amniotic membrane				√							
	Umbilical cord				√							
Vaginal swab	Vaginal swab			√	√							
Breast milk	N/A				√	√	√	√	√	√	√	
Oral sample	Mouth rinse		**√**		√							
	Buccal swab		**√**		√							
Nose	Nasopharyngeal swab		**√**					√	√			
Skin	Skin swab (forearm)		**√**					√	√			
Hair	N/A		**√**					√	√			
Nail	N/A		**√**					√	√			
Father			1									
Urine	N/A		√									
Stool	Raw/Norgen stool preservative		√					√				
Oral sample	Mouth rinse		√									
Hair	N/A		√									
Nail	N/A		√									
Infant												
Stool	Fresh/Norgen stool preservative				√	√	√	√	√	√	√	√
Oral sample	Buccal swab				√	√	√	√	√	√	√	√
Hair	N/A							√	√	√	√	√
Nail	N/A							√	√	√	√	√
Nose	Nasopharyngeal swab					√	√	√	√	√	√	√
Skin	Skin swab (forearm)					√	√	√	√	√	√	√
Environment								√				
Air	Swab				√	√^2^						

^1^ Father's baseline bio‐samples were collected on random days during mother's pregnancy.

^2^ Air swabs at pediatric clinics were collected at random follow‐up during 1–12 months.

**Table 2:** Overview of measurement in questionnaires.

Assessment	Validated instrument	Pregnancy		Birth/before discharge	Neonatal (months)		Infancy (months)				Childhood (years)
		Baseline (T1/T2/T3)	T2/T3		1	2–3	6	12	18	24	3–7
Parental status											
Diet											
Early life (0–18 years) dietary habits		√#									
Dietary preference^2^		√#									
Current food additive intake	FAQ	√#					√		√		
Current (In the past 3 months) dietary habits	FFQ						√		√		
Synbiotic/probiotic intake		√	√		√	√	√	√	√		
Life style											
Smoke		√#	√		√	√	√	√	√		
Alcohol intake		√#	√		√	√	√	√	√		
Physical exercise in the past week		√#	√		√	√	√	√	√		
Health status											
Self‐administrate disease report		√#	√^3^	√^4^	√^3^	√^3^	√^3^	√^3^	√^3^		
Disease status change (by OG nurses)					√	√	√	√	√		
Psychosocial status	PHQ‐9		√		√	√	√	√	√		
Stool type	BSC	√#	√		√	√	√	√	√		
Baby status											
Neonatal complications				√							
Delivery status	APGAR			√							
Self‐administrate health report (by the mother)				√^	√^3^	√^3^	√^3^	√^3^	√^3^	√^3^	√^3^
Disease status change (by pediatric nurses)					√	√	√	√	√	√	√
Skin care^5^					√	√	√	√	√	√	√
Feeding status^6^				√	√	√	√	√	√	√	
Detailed current (in the past day) food intake	FQ						√		√	√	√
Sleeping status^7^					√	√	√	√	√	√	
Eating behavior	CEBQ									√	√
Early Childhood Behavior	ECBQ									√	√
Pediatric follow‐up					√	√	√	√	√	√	

Abbreviations: APGAR, Apgar Score; BSC, Bristol Stool Chart; CEBQ, Child Eating Behavior Questionnaire; ECBQ, The Early Childhood Behavior Questionnaire; EDTA, Ethylenediaminetetraacetic Acid; FAQ, Food Additive Questionnaire; FFQ, Food Frequency Questionnaire; PHQ‐9, Patient Health Questionnaire‐9.

^#^ Father information

^2^ Prefer meats or vegetables, is he/she vegetarian, vegan, or on gluten‐free/lactose‐free/low‐carbohydrate/low‐sodium/low‐calorie/high‐protein diet

^3^ Only the newly occurred health issue(s) would be recorded

^4^ Pregnancy/obstetric labor complication

^5^ Including time of last bath, use of soap/lotion (and brands), skin problems, and the medication for the skin problems (if any)

^6^ Including feeding (Breastfeeding/formula milk/mix) mode, breastfeeding mode (Direct/pump/mix), frequence, stop day of breastfeeding (if any), and brand of formula milk

^7^ Including total sleeping length, awaking length, activities within 1 h before sleep, accompany(ies), and overall quality

The primary objective of the MOMMY cohort is to study early‐life mother‐to‐infant microbial transfer and the effect of such microbial colonization on infants' later‐life development and responses.

The secondary objectives include:

(1) to establish a metagenomics platform and framework to study vertical microbial transmission from mothers to babies,
(2) to investigate the changes in the microbiome in infants born to mothers with different health conditions,
(3) to define the healthy early life microbiome in the first 1000 days in the Chinese population,
(4) to study the correlation and/or causation between the microbial transmission patterns and the short and medium‐term health and disease of babies, including neurodevelopment, growth, allergies, and infectious diseases.

### How to select the representative study sites?

In MOMMY, we specifically aimed to examine the differences between regional (urban vs. rural) lifestyle and their impacts on the vertical transmission of microbiota. Urban lifestyles have been associated with increased risk for “Western” diseases and the pathogenesis of such diseases related to gut microbiome dysbiosis [18]. However, the effects of urban lifestyle on the early life microbiome remain unknown. Here, we incorporated a well‐established urban location, a recently expanded industrialized population, and a third urban site predominated by rural‐to‐urban populations. Hong Kong (**Study site 1**) is a highly urbanized and densely populated metropolitan city. Foshan (**Study site 2**) is an equally important part of the Greater Bay Area as a national base for advanced manufacturing and Guangdong's key production hub. In recent years, it has upgraded and transformed its manufacturing industry, ranking first in industrial technology transformation investments in Guangdong Province for four consecutive years. Foshan was also one of the first in national demonstration base for agricultural industrialization [https://www.bayarea.gov.hk/en/about/foshan.html]. In contrast, Yunnan is a large province in China consisting of both urban and rural residents. Kunming (**Study site 3**), the provincial capital of Yunnan, is an urban city with a multi‐ethnic and diverse population originating from rural districts spanning Yunnan [19, 20, 21]. Furthermore, these cohorts are stratified by their economics, with high incomes in Study Site 1 and lower incomes in Study Sites 2 & 3 (Figure 1B).

Prenatal maternal/paternal diet is another major determinant of the lifespan of offspring and future health and disease outlook [22]. Ultra‐processed foods and food additives dominate the global food system, with up to 50% of daily calories consumed in high‐income countries and decreasing with economic status [23]. This phenomenon was observed in our cohort, with the overall food additive intake decreasing from the high‐income area (Hong Kong) to lower‐income areas (Foshan and Kunming) (Figure 1B) [24]. Accumulating evidence suggests that food additives can lead to altered gut microbiome composition [23, 25, 26, 27]. Given these circumstances, we anticipate the rapid economic development and social transition occurring in Kunming, Foshan, and Hong Kong are likely to be replicated in other places in China in the near future, leaning weight to the importance of these studies. Moreover, the unique population distribution and comprehensive phenotype data allow us to dissect the impacts of geography, urbanization, diet, and host‐related factors on early life microbiome configuration, as well as functional insights from multi‐omics in a well‐defined early life Chinese population. Thus, MOMMY will inform and guide researchers, policy makers and other stakeholders in these areas with insights specific to our population needs.

**Figure 2:**
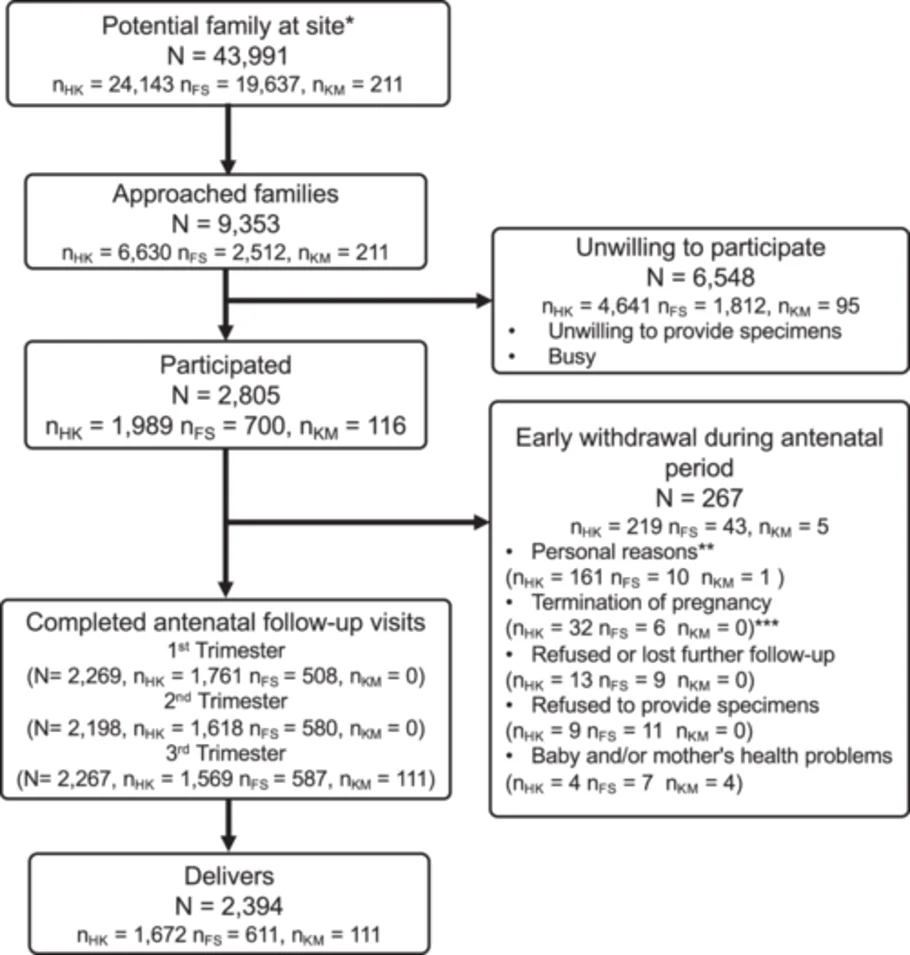
Flow chart for subject recruitment and follow‐up in the MOMMY cohort (up to 31/03/2024). *N*: total sample size, *n*_HK_: sample size in Hong Kong, *n*_FS_: sample size in Fo Shan, *n*_KM_: sample size in Kun Ming. * Study sites: Prince of Wales Hospital, the First People's Hospital of Foshan, the First Affiliated Hospital of Kunming Medical University. ** Personal reasons: busy, immigration, inconvenient location for follow‐up, other family members' disagreement, other personal reasons. *** Termination of pregnancy: abortion or miscarriage.

Figure 2 shows the overview of the study design, which is currently composed of three regions in China (Hong Kong, Foshan, Kunming), a total of three hospitals, including Prince of Wales Hospital (a regional government public hospital located in Sha Tin, New Territories in Hong Kong), the First People's Hospital of Foshan (founded in 1881 with 3A‐grade hospitals in China) and the First Affiliated Hospital of Kunming Medical University (the No. 1 School of Clinical Medicine of Kunming Medical University, established in 1941 and approved among the first batch of 3A‐grade hospitals in China) that were voluntarily enrolled.

### How to place MOMMY in the world map of global early life microbiome research?

**Figure 3:**
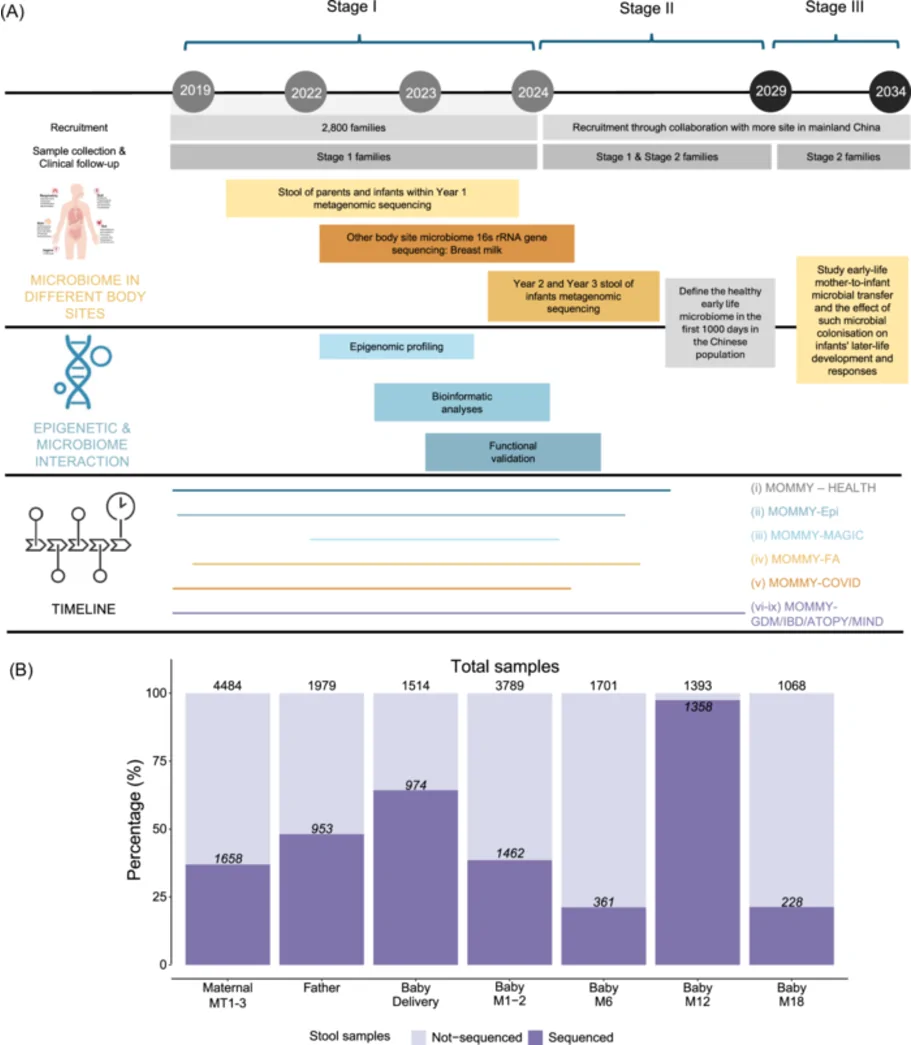
MOMMY cohort stage and stool metagenomic sequence progress. (A) The MOMMY will include three stages. (B) Stool metagenomic sequence progress.

The MOMMY will include three stages (Figure 3A). The study was approved by the Institutional Review Board of all participating centers. The Human Genetic Resources Administrative Permit application is being prepared and will be secured before any genetic analysis. Stage I (September 2019‐September 2024), which was an initial phase to manage infrastructure, recruit subjects and build an integrative microbiome analysis platform to standardize reproducible body‐wide microbiome SOPs for sampling, data generation, and computational methods for microbiome analysis and epidemiology interpretation. Despite the challenges of the COVID‐19 pandemic, we were able to recruit around 2800 families, resulting in 2500 births in Hong Kong, Foshan, and Kunming. Amongst these families, over 130,000 biological samples were collected, including stool, maternal/cord blood (for buffy coat, serum, and plasma [EDTA]), placenta, urine, vaginal swab, buccal swabs, breastmilk, nasal swabs, hair, and nail) and >30,000 web‐based clinical metadata that included dietary habits and lifestyle data based on validated questionnaires from parents and their infants at multiple time points in the antenatal, delivery and postnatal period. Apart from the clinical progress, we also performed large‐scale in‐house metagenomic sequencing on stool samples. Infants were followed up to 3 years of age with over 80% retention rate in three sites, especially the Hong Kong site (Figure 2). Interestingly, five nationalities (Chinese, Canadian, Korean, American, Malaysian, and 99.9% were Chinese) and 13 ethnicities (Han, Hui, Huang, Mang, Tujia, Buyi, She, Miao, Dong, Man, Yao, Amei, Yue) are in MOMMY to date (Table 3). Although the sample size of ethnic minorities is limited, we may conduct pilot studies to explore the uniqueness of lifestyle, epigenetics, and microbiome in these specific populations and may expand sub‐studies according to the relevant research aims.

**Table 3:** Characteristics among three MOMMY study sites.

Characteristic^1^	Hong Kong Female *N* = 1465	Hong Kong Male *N* = 1059	Fo Shan Female *N* = 528	Fo Shan Male *N* = 503	Kun Ming Female *N* = 107	Kun Ming Male *N* = 102
Age at baseline	32.8 (30.4, 35.8)	34.4 (31.7, 37.9)	30.8 (28.3, 33.1)	31.9 (29.5, 35.0)	29.3 (26.9, 32.5)	31.8 (28.9, 34.2)
NA	6	0	0	0	3	3
BMI	21.0 (19.4, 23.2)	24.0 (21.8, 26.4)	20.4 (18.9, 22.3)	23.7 (21.5, 25.8)	20.8 (19.2, 22.8)	23.2 (22.1, 24.7)
NA	73	0	4	0	0	1
BMI Group^2^						
Underweight (<18.5)	822 (59%)	352 (33%)	320 (61%)	182 (36%)	66 (62%)	47 (47%)
Healthy weight (18.5–23)	184 (13%)	43 (4.1%)	100 (19%)	21 (4.2%)	17 (16%)	1 (1.0%)
Overweight or obese (>23)	386 (28%)	664 (63%)	104 (20%)	300 (60%)	24 (22%)	53 (52%)
Ethnicity						
Han	1,422 (98%)	1,043 (99%)	522 (99%)	499 (99%)	100 (93%)	100 (98%)
Other	36 (2.5%)	15 (1.4%)	6 (1.1%)	4 (0.8%)	7 (6.5%)	2 (2.0%)
NA	7	1	0	0	0	0
Marital status						
Married	1,382 (95%)	‐‐‐	525 (99%)	‐‐‐	107 (100%)	‐‐‐
Single/widowed/divorced	75 (5.1%)	‐‐‐	3 (0.6%)	‐‐‐	0 (0%)	‐‐‐
NA	8	‐‐‐	0	‐‐‐	0	‐‐‐
Any older sibling (s)	472 (32%)	‐‐‐	201 (38%)	‐‐‐	57 (53%)	
NA	9	‐‐‐	0	‐‐‐	0	‐‐‐
Education						
High school or below	427 (29%)	340 (32%)	39 (7.4%)	65 (13%)	27 (25%)	27 (26%)
Bachelor or equivalent	868 (60%)	565 (53%)	440 (83%)	386 (76%)	62 (58%)	53 (52%)
Master or above	162 (11%)	154 (15%)	49 (9.3%)	54 (11%)	18 (17%)	22 (24%)
NA	8	0	0	0	0	0
Working status						
Full‐time	1,121 (77%)	987 (93%)	461 (87%)	8 (1.6%)	76 (71%)	102 (100%)
Part‐time	53 (3.6%)	34 (3.2%)	11 (2.1%)	481 (96%)	3 (2.8%)	0 (0%)
Housewife or other^3^	284 (19%)	38 (3.6%)	56 (11%)	12 (2.4%)	28 (26%)	0 (0%)
NA	7	0	0	2	0	0
Individual income						
<5000	117 (10%)	11 (1.2%)	70 (15%)	41 (8.4%)	24 (35%)	22 (26%)
5000–14,999	143 (12%)	41 (4.4%)	373 (80%)	317 (65%)	43 (63%)	62 (74%)
15,000–29,999	518 (45%)	370 (40%)	19 (4.1%)	102 (21%)	1 (1.5%)	0 (0%)
≥30,000	385 (33%)	513 (55%)	6 (1.3%)	28 (5.7%)	0 (0%)	0 (0%)
NA	302	124	60	15	39	18
Tobacco smoking						
Never	1,339 (92%)	781 (74%)	521 (99%)	356 (71%)	103 (96%)	54 (53%)
Past (Before recruitment)	119 (8.2%)	123 (12%)	7 (1.3%)	34 (6.8%)	4 (3.7%)	5 (4.9%)
Current (At recruitment)	0 (0%)	155 (15%)	0 (0%)	113 (22%)	0 (0%)	43 (42%)
NA	7	0	0	147	0	48
Alcohol use^4^	540 (37%)	569 (54%)	157 (30%)	340 (68%)	25 (24%)	54 (53%)
NA	7	0	0	0	1	0
Physical exercise/week						
0	1,101 (76%)	499 (47%)	514 (97%)	312 (62%)	100 (93%)	50 (49%)
1–2 day(s)	248 (17%)	367 (35%)	8 (1.5%)	129 (26%)	7 (6.5%)	30 (29%)
3 or above day (s)	108 (7.4%)	193 (18%)	6 (1.1%)	62 (12%)	0 (0%)	22 (22%)
NA	8	0	0	0	0	0
Dietary habit						
Vegetable > meat	366 (27%)	80 (8.1%)	208 (39%)	75 (15%)	21 (20%)	5 (5.0%)
Vegetable = meat	566 (42%)	329 (33%)	220 (42%)	226 (45%)	79 (75%)	69 (68%)
Vegetable < meat	406 (30%)	580 (59%)	100 (19%)	202 (40%)	5 (4.8%)	27 (27%)
NA	127	70	0	0	2	1
Any disease (s)	391 (27%)	263 (25%)	353 (67%)	329 (65%)	38 (37%)	2 (2.0%)
Digestive system (%)^5^	391 (27%)	263 (25%)	112 (21%)	108 (21%)	38 (37%)	2 (2.0%)
IBD (%)	45 (3.1%)	39 (3.7%)	3 (0.6%)	5 (1.0%)	14 (13%)	1 (1.0%)
IBS (%)	10 (0.7%)	2 (0.2%)	3 (0.6%)	5 (1.0%)	11 (10%)	0 (0%)
Immune system (%)^6^	5 (0.3%)	2 (0.2%)	5 (0.9%)	6 (1.2%)	0 (0%)	0 (0%)
Allergy (%)^7^	11 (0.8%)	8 (0.8%)	28 (5.3%)	19 (3.8%)	1 (0.9%)	0 (0%)
Eczema (%)	191 (13%)	126 (12%)	105 (20%)	99 (20%)	3 (2.9%)	0 (0%)
Heart disease (%)^8^	17 (1.2%)	20 (1.9%)	6 (1.1%)	9 (1.8%)	0 (0%)	0 (0%)
Liver disease (%)^9^	13 (0.9%)	6 (0.6%)	3 (0.6%)	5 (1.0%)	1 (0.9%)	0 (0%)
Renal disease (%)^10^	13 (0.9%)	22 (2.1%)	38 (7.2%)	111 (22%)	1 (0.9%)	0 (0%)
Reproductive system (%)^11^	1 (<0.1%)	0 (0%)	15 (2.8%)	17 (3.4%)	0 (0%)	1 (1.0%)
Hyperglycemia/DM (%)^12^	29 (2.0%)	4 (0.4%)	80 (15%)	9 (1.8%)	12 (11%)	0 (0%)
Hypertension (%)	20 (1.4%)	5 (0.5%)	20 (3.8%)	5 (1.0%)	1 (0.9%)	0 (0%)
Hyperlipidemia (%)	15 (1.0%)	23 (2.2%)	3 (0.6%)	10 (2.0%)	0 (0%)	0 (0%)
Thyroid disease (%)	8 (0.5%)	7 (0.7%)	5 (0.9%)	43 (8.5%)	0 (0%)	0 (0%)
Cancer (%)	52 (3.6%)	7 (0.7%)	65 (12%)	18 (3.6%)	7 (6.5%)	0 (0%)
Psychological disorders (%)	1 (<0.1%)	5 (0.5%)	4 (0.8%)	4 (0.8%)	0 (0%)	0 (0%)
Other disease (%)	19 (1.3%)	5 (0.5%)	4 (0.8%)	1 (0.2%)	0 (0%)	0 (0%)
Self‐report disease (s) NA	127	70	0	0	2	1

*Note*: NA = 0 unless specified.

Abbreviations: BMI, body mass index; IQR, interquartile range.

^1^ Age: Median (IQR); Others: *N* (%)

^2^ BMI (Body Mass Index) group: mothers' BMI group refers to their pre‐pregnancy BMI

^3^ Other working status: Student and unemployed

^4^ Mother's alcohol intake refers to the status before pregnacy

^5^ Including following options: gluten Intolerance, inflammatory bowel disease (IBD), irritable bowel syndrome (IBS), lactose intolerance, polyps/cancer, piles, gastritis or Gastric ulcer, or others.

^6^ Including following options: rheumatoid arthritis, systemic lupus erythematosus, psoriasis, or others.

^7^ Including following options: asthma, atopic dermatits/eczema, food allergy, allergic rhinitis, or others.

^8^ Including following options: coronary atery disease or others.

^9^ Including following options: hepatitis B carrier, other hepatitis, fatty liver, and others.

^10^ Including following options: glomeronephritis, diabetic nephropathy, or others.

^11^ Including following options: polycystic ovarian syndrome, or others.

^12^ Including following options: gestational diabetes, prediabetes (slightly high blood sugar, but not diabetes), type 1 diabetes, type 2 diabetes and age of onset).

During Stage II (September 2024–September 2029), the 2800 families recruited during the first stage will be further followed up till 7 years old of children. Specifically, MOMMY will develop the management algorithm and tools to define the healthy early life microbiome in the first 1000 days in the Chinese population, identify predictors for childhood allergy atopy march and neurodevelopmental disorder (NDD) diseases, and offer early intervention and prevention. Besides, it will be an implementation stage to expand up to 20,000 family pairs (mother, father, and their newborns) and collect the world's largest cohort of 100,000 maternal, paternal, and fetal samples through collaboration with hospitals and partners in mainland China, such as the northern or central cities of China.

In Stage III (September 2029–September 2034), the large‐scale families recruited during Stage II will follow up and further investigate the childhood disease. In this phase, we will focus on children in the critical developmental age of 5 to 7 years. This age group is particularly vulnerable to various adverse conditions and physiological states, such as NDDs and obesity, which require targeted interventions to promote their well‐being and development. By targeting a subgroup of infants, we aim to create effective microbiome interventions that address the adverse conditions faced by children. MOMMY will scale up and establish an Alliance of Early Life Microbiome to foster interdisciplinary efforts to reach new horizons.

### How to standardize the procedure of the early life microbiome‐related birth cohort?

#### Eligibility and enrollment

To disseminate and advertise the MOMMY cohort, the promotional videos and posters were displayed at antenatal clinics and related websites in three substudies sites. Interested subjects at any stage of gestation were invited to participate. Participants are included if they are pregnant and have plans to give birth at a local hospital, plan to stay in the same local area for at least 7 years post‐delivery, and are competent to provide informed consent. Biological fathers are also invited to participate. There are no other exclusion criteria, except for cases of miscarriage or termination of pregnancy, which will be withdrawn from the study. Babies are included in the birth cohort on the date of birth. Research assistants or research nurses will provide additional detailed information about the biological samples and metadata collection of the study and explain the informed consent form. All subjects can withdraw from the study at any time without jeopardizing their standard care.

#### Procedures for biological samples collection, processing and storage

Biobanks or biorepositories are facilities that collect, process, store and distribute clinical samples and associated data, mainly for biological and medical research. A well‐established quality check and assurance system is essential to a Biobank, which will positively improve the sample quality and integrity. This will also ensure the credibility, consistency, reliability, and reproducibility of research data that is generated from utilizing these clinical samples.

An overview of all biological samples collected in the study is shown in Table 1. Stool and urine samples are collected from the mother during the antenatal to postnatal period at the pre‐specified time points. Biological samples during delivery are collected, including venous blood, cord blood, placenta, vaginal swabs, and air swabs. The air swabs are collected from two primary locations: the delivery ward (for vaginal deliveries) and the operating theater (for cesarean sections) and the air swabs were stored in two separate cryotubes. Other maternal biological specimens, including breastmilk (collected simultaneously with postnatal stool and urine samples), oral samples, nasopharyngeal swabs, skin swabs, hair, and nails, are also collected during the antenatal period once and twice after delivery. Stool, urine, nails, hair, and oral samples are collected twice from the father. For newborn babies, meconium, stool nails, hair, buccal swabs, nasopharyngeal swabs, and skin swabs are collected longitudinally at specified intervals.

To ensure consistency of sample collection, the MOMMY biobank research team provides the SOP for the collection and submission of samples to the participants. Except for stool and urine samples, all samples are collected by nurses during our follow‐up clinics, stored in a 4°C freezer, and sent back to the MOMMY biobank laboratory for aliquoting processing within 24 h. Biological samples during delivery are collected by trained nurses and stored in a refrigerator at 4°C. Nurses will notify the MOMMY team to collect samples as soon as possible and deliver the samples to the MOMMY laboratory and biobank, where samples are processed and then stored at −80°C for further research (Laboratory Standard Operating Procedures (LSOP)). This LSOP provides detailed steps for the pickup, processing, and storage of each sample type under the project MOMMY. It aims to provide the MOMMY team members with a stepwise approach to process the samples that will meet the standards for downstream research needs. This LSOP, however, does not cover detailed safety procedures for handling clinical samples, and it is recommended that personnel follow institutional biosafety guidelines.

#### Measurements of the parents and babies

Extensive online self‐administered questionnaires, validated questionnaires, and research notes recorded by the research teams during follow‐up visits will be used to obtain metadata. First, baseline demographics will be comprehensively documented, including the parents' diets, lifestyles, health status, and psychosocial status (Tables 1, 2). In regard to parental diet, parents' early life (0–18 years old) approach to processed foods, current intake of food additives, and dietary preferences are collected at baseline [28]. The parents' prebiotic and probiotic intake is recorded at each follow‐up, while the food frequency questionnaire (FFQ)‐based food additive consumption questionnaire for the parents is collected at baseline, postnatal months 6 and 18. For parental lifestyles, we recorded data related to maternal smoking status, alcohol intake, and physical exercise during the past week at each follow‐up time point. Paternal information was also recorded at baseline and 6 months after childbirth. Psychosocial status is measured with Patient Health Questionnaire‐9 (PHQ‐9) and Edinburgh Postnatal Depression Scale since the third trimester and at 6 months post‐delivery for mothers [29]. Stool types are recorded at each follow‐up by providing the Bristol stool form scale (BSC) with schematic drawings of different levels of stool [30] (Table 2).

Regarding the infants, the health information is recorded at birth, including any antenatal complications, Apgar score (at 1 min and 5 min), any use of assisted ventilation at birth, any severe complications and the treatment, and any special care baby unit or neonatal intensive care unit admission and the length of stay. Postnatal self‐administered health reports are provided by the mother, and disease status changes are collected by pediatric research nurses during follow‐up at months 2, 6, 12, and 18. Additionally, the feeding status and dietary pattern were collected at delivery, months 2, 6, 12, and 18, and the FFQ of infants will also be measured at months 6 and 18. The early childhood behavior will be measured with The Early Childhood Behavior Questionnaire at 2–7 years old [31] (Table 1).

### How to follow‐up and retain subjects in early life microbiome‐related birth cohort?

#### Follow‐up and retention strategies

Comprehensive strategies are employed to retain participants throughout the study. During the antenatal period, mothers can contact the research team for any pregnancy‐related issues, with unscheduled research visits arranged as necessary. Post‐delivery, mothers often have numerous questions regarding breastfeeding and their babies' health; therefore, a dedicated hotline is established to provide professional nursing advice. Additionally, we offer free health checks, including a skin prick test in year two and an eye test in year three. Regular seminars on topics such as healthy diet, autism, and eczema are organized for families. During these seminars, we inquire about the child's eating habits and provide brief on‐site consultations for any noticeable eating problems. Furthermore, children with concerning standard health check results between 1.5 and 3 years old are offered an in‐depth interview for early assessment of behavioral issues. Notably, we also provide a microbial report for all 1‐year‐old infants at the Hong Kong site, allowing parents to arrange a consultation with the research team if necessary.

Figure 2 shows study participant recruitment and follow‐up until 31st March, 2024. At this stage of recruitment, a total of 2805 mother‐baby pairs (Hong Kong: 1989, Foshan: 700, Kunming: 116) were recruited, with a drop rate of 9.5% (267/2805) had withdrawn consent for participation during antenatal period, with the following reasons: (1) Personal reasons (64.4%), (2) Termination of pregnancy (14.2%), (3) Refused or lost further follow‐up (8.2%), (4) Refused to provide specimens (7.9%), and (5) Baby and/or mother's health problems (5.6%). During the postnatal (at any time point from delivery to postnatal year three) period, a total of 363 mother‐baby pairs were withdrawn (15.2% 363/2394). Specifically, we reached a response rate (with sample/questionnaire collected) of (2198/2394, 91.8%) at trimester two, (2267/2394, 94.7%) at trimester three.

### How to get robust clinical and microbiome data post‐collection?

#### Quality control and data management

Extensive online questionnaires for self‐administration and research notes recorded by the research teams during follow‐up visits will be used to obtain metadata, including demographic data, dietary habits, lifestyle, health supplements, health conditions, medications, and psychosocial status for mothers, fathers, and babies. In the two study sites in mainland China, paper questionnaires will be used initially, with clinical teams later entering the data into an encrypted database. Co‐morbid illnesses and medications will be cross‐checked against the hospital management system, which covers 90% of the out‐patient clinic follow‐up for the entire population. (1) Queue Maintenance and Control of Missed Visits: Enrolled families will be scheduled for more than 20 follow‐up visits from delivery to the child's age of 7 years. One week before each scheduled visit, a reminder call will be made. If a family misses a visit, the clinical team will contact the parents to reschedule within the designated time window. If an in‐person follow‐up is impossible, research teams will conduct a phone interview. After each clinic visit, an individual electronic questionnaire link will be sent via email or social media apps, with parents asked to complete the questionnaires within 2 weeks. A reminder message will be sent if the questionnaires are not completed on time. (2) Quality Control and Data Monitoring: SOPs are established for each stage to ensure data quality and integrity, including study teams' on‐site instructions for completing questionnaires and collecting specimens during antenatal and postnatal visits, guidelines for parents on electronic questionnaire completion, first‐stage data checking, data monitoring, and data set compilation. Data will be saved in a secure, password‐protected database managed by the Chinese University of Hong Kong. The questionnaire data and research notes will be downloaded every 3 months for monitoring. Initially, one member of the monitoring team will review the data set for completeness and errors according to the SOP. Any problematic records will be returned to the clinical team, who will contact parents for corrections. If the data are time‐sensitive, extra attention will be required to ensure accuracy. After the first review, another monitoring team member will recheck the data for quality and logical consistency. Any issues will be annotated and returned to the clinical team for further follow‐up. Queried data will be verified against the hospital management system, and telephone calls will be made if necessary. The updated data set will then undergo a final verification by the monitoring team. Each verified data set will be assigned a batch number, and an independent database team member will compile different batches according to the SOP to maintain data alignment.

#### Robust methodology to handle missing data

After the data management and monitoring by the clinical team for those key variables that maintain a missing rate of more than 30%, the complete case analysis will be conducted [32]. For those longitudinal co‐variates (smoking, alcohol drinking, disease, long‐term medication, chronic disease), the Last Observation Carried Forward methods will be applied to replace the missing data [33]. For data that belong to missing completely at random or missing at random, multiple imputation will be used for data imputation for other constant variables (Baseline characteristics including education, working status, incomes, etc.) or anthropometry data (childhood/infancy height, weight, head circumference) with a missing rate of less than 30% [34].

#### The standard operating procedure (SOP) for metagenomic sequence

DNA extractions of stool samples will be carried out using the Qiagen DNeasy PowerSoil Pro Kit protocol (Qiagen, Cat#47016, 250 reactions). All operations will be completed in the biosafety cabinet (BSC). In parallel, positive control (a microbial community standard (ZYMO, Cat#D6300)) and three negative controls (BSC environment negative control, operation extraction negative control, and preservative solution negative control) will be included for each kit as well as each extraction batch. DNA quality will be checked using gel electrophoresis to determine DNA shearing and using a Nanodrop OneC Spectrophotometer to determine DNA quality. Qualified DNA samples will be stored at −80°C for further library construction. Extracted DNA concentrations will be measured using Qubit (ThermoFisher) with the Qubit dsDNA BR Assay Kit (ThermoFisher). Illumina sequencing libraries will be prepared from 1 to 300 ng DNA using the Illumina DNA Prep kit according to the manufacturer's recommended protocol, with reaction volumes scaled accordingly. Additionally, Nuclease‐Free Water (not DEPC‐Treated) (Invitrogen™) will be utilized as one negative control, and ZymoBIOMICS Microbial Community DNA Standard (Catalog #: D6306) will be constructed for one positive control. The library concentration is determined by the Qubit dsDNA HS Assay Kit (ThermoFisher), while the quality of the library is checked by analyzing pooled libraries using the Agilent 2100 Bioanalyzer with a High Sensitivity DNA kit to determine the average fragment size of 600 bp. Libraries will be sequenced on the Illumina Novaseq. 6000 platform with 150 bp paired‐end reads. For sequence depth, a sequencing depth of round 5 gigabase (Gb) per fecal sample is aimed. And it is worth further adjustments based on preliminary data outcomes. For sequencing quality, quality score Q30 is used to assess the quality of sequencing reads [35]. The samples with Q30 (%) higher than the quality threshold that Illumina officials provided (≥85% of bases higher than Q30 at 2 × 150 bp for NovaSeq. 6000 System, https://www.illumina.com/systems/sequencing-platforms/novaseq/specifications.html) will be considered as qualification. To mitigate the risk of longitudinal metagenomic sequencing, which may introduce batch effects, during the metagenomic sequencing phase, we are implementing proactive quality control measures in our wet‐labaratory procedures. Specifically, each batch of DNA extraction and library construction will include both a Positive Technical Control (PTC, consisting of a mock microbial community) and a Negative Control (NTC, a no‐template control). The PTC serves as a benchmark to quantify technical variation across batches, while the NTC is used to monitor potential contamination. This approach provides an empirical measure of batch‐specific technical noise and establishes an objective basis for subsequent computational adjustments. Additionally, to further minimize batch effects across different stages, we will employ computational tools designed to correct for such artifacts. Methods such as conditional quantile regression (ConQuR), negative binomial regression models, or the multivariate integration method will be applied as appropriate to ensure robust and reproducible results [35, 36, 37].

#### Analytical strategy for integrating multi‐omics data processing of biological samples

Fecal to assess the role of early‐life microbiome in the development of health, and how gut microbiome dysbiosis could contribute to disease development. Meanwhile, feacal calprotectin, metabolomics, or sweeteners could be assessed using fresh stool. Breast milk samples will be analyzed using untargeted and targeted metabolomics sequencing for their human milk oligosaccharides content and small molecules. Umbilical cord blood will be prepared to evaluate the transfer of maternal metabolites and immune molecules to the infant, and how it may establish and maintain health development in children. Vaginal swabs and maternal oral samples are collected to investigate the sources of neonatal gut microbiome. Air swabs of the hospital room during delivery are collected for further analysis of environmental exposure to the neonatal microbiome. Nasopharyngeal swabs from infants are collected to investigate the relationship between nasopharyngeal microbiomes and the development of their physical health and respiratory disease. The hair and nail samples are collected for further DNA analysis, extending the host genomic analysis from single genes to genome‐wide analysis to investigate how host genetics shape early life microbiota development. Furthermore, collecting and storing cord blood and placentas grants us the opportunity to delineate how human genetics influence early life microbiome composition.

For other body sites with 16S/ITS data, ASV‐level transmission rates will be calculated with the stool metagenomic to match the corresponding resolution. Personal mother‐infant transmission events will be assessed by calculating narrow‐sense heritability (h2 defined as the proportion of variance in microbial presence/absence profiles explained by additive genetic effects, which will also capture the shared exposure [38]. These data will help to answer which body site contributes to the infants' gut microbiome. And whether the proportion of the microbiome sourced from other body sites, besides the gut, affects the health and disease of infants.

Stool metagenomic and metabolomic data, skin/nasopharyngeal swab/breastmilk 16S rRNA amplicon sequencing data, and blood epigenetic data will be considered as different dimension data, and therefore the multi‐omics models will be applied. To deal with such high‐dimensional omics data, our analytical strategies varied. For those samples with omics assessments, multiblock Projection to Latent Structures will be conducted for either unsupervised or supervised analysis or Canonical Correlation Analysis will be employed according to the study aims (for classification or regression).

#### Strain resolution techniques to study microbiome transmission

Strain characterization will be performed using StrainPhlan based on pre‐processed metagenomic data to further characterize the single‐nucleotide variants (SNVs) [39]. Strain transmission rates will be calculated as the number of strain transmissions between two samples divided by the number of shared species profiled by StrainPhlAn (number of transmission strains/number of shared species). Strain acquisition rates by the offspring will be defined as the proportion of strains profiled in the offspring transmitted from mothers, thus putatively originating from them. Species transmissibility will be defined as the number of strain transmission events detected for a species divided by the total potential number of strain transmission events based on the presence of a strain‐level profile by StrainPhlAn [40]. We will mainly assess strain transmission across the following modes: related mother‐infant (defined between mothers and their offspring). The transmission between mother‐infant pairs will be evaluated using mothers' stool samples during late pregnancy, which is the closest to delivery time. Further, we will perform de novo assembly and Metapi will be applied [41]. Briefly, the high‐quality reads will be assembled using MEGAHIT [42]. To facilitate genome recovery, reads of samples from the same infants collected at multiple time points will be co‐assembled. Reads will be mapped back to the resulting contigs using minimap with default parameters [43]. Binning of contigs into metagenome‐assembled genomes (MAGs) will be done using two automatic binning programs: MetaBAT2 and VAMB [44, 45]. High‐ (completeness > 90% and contamination < 5%) or medium‐quality (completeness > 50% and contamination < 10%) MAGs (MAGs) will be identified by CheckM, consistent with Minimum Information about a Metagenome‐Assembled Genome (MIMAG) standard [46, 47]. The taxonomic assignment of these representative bacterial species‐level MAGs will be performed by GTDBTk based on the GTDB database [48, 49]. InStrain will be applied to sensitively detect shared microbial strains based on MAGs of species [50]. Apart from the stool samples, the placenta sample and one air swab sample collected during delivery will help to disentangle the true microbial transfer effects from the shared environment between mother and infant.

#### Move forward from correlation to causality

To infer causality from our large‐scale cohort data, we will employ two complementary statistical approaches: (i) Mediation Analysis [51, 52]: We will perform formal causal mediation analysis using structural equation modeling to test whether specific microbial taxa mediate the effect of an exposure (e.g., mode of delivery, diet) on a health outcome (e.g., allergic sensitization, growth metrics). This framework will help delineate potential causal pathways (e.g., Cesarean section → reduced Bifidobacterium → increased asthma risk). (ii) Two‐Sample Mendelian Randomization (MR): We will leverage genetic instruments from large‐scale genome‐wide association studies (GWAS) [52, 53] of gut microbiome traits as proxies for microbial abundance. By applying these instruments to outcome GWAS data, we can estimate the causal effect of the microbiome on child health outcomes while controlling for confounding variables.

Statistical inference will be directly tested and validated through controlled animal experiments designed to establish mechanistic causation. (i) Fecal microbiota transplantation: We will transplant fecal samples from well‐phenotyped MOMMY cohort infants (e.g., from infants who developed atopy vs. those who remained healthy) into germ‐free or antibiotic‐preconditioned mice. A successful transfer of phenotype (e.g., immune dysregulation) would provide direct evidence that the microbial community is causal. (ii) Mono colonization and Defined Consortium Assays: To move beyond community‐level associations and identify specific effector strains, we will: Monocolonize germ‐free mice with individual bacterial species isolated from vertically transmitted lineages (e.g., specific *Bifidobacterium* or *Bacteroides* strains) to assess their unique impact on host immune development. Assemble and administer a defined microbial consortium comprising core vertically transmitted species. This consortium will be used to colonize mice to test the synergistic effect of these strains on promoting early‐life immune maturation and protecting against disease challenges, a strategy that has been employed to induce regulatory T cells [54]. iii) Addressing Translational Feasibility (Colonization Resistance): We acknowledge that colonization resistance is a critical barrier for clinical translation. Ecological principles will be considered in consortium design. For specific pathogen‐free mice, we will precondition mice with antibiotics or use native strains with high fitness and a history of vertical transmission to enhance engraftment success.

## 3 STUDIES DERIVED FROM MOMMY

### MOMMY inception cohort: Research novelty and projections

In the MOMMY inception cohort, biosamples including stool, breastmilk, vaginal swab, skin swab, maternal and cord blood have been prospectively collected from 1200 Hong Kong families (mothers, fathers, and offsprings) from pregnancy until the birth of infants followed up to first years of age as of the end of March 2024. In total, >10,000 stool samples from the MOMMY inception cohort families (mother, father, and their infants who reached 1 year old) have been performed in metagenomic sequence (Figure 3B). Leveraging the standardized reproducible body‐wide microbiome sampling SOP, data generation, and computational methods for microbiome analysis and epidemiology interpretation, we will be able to build this platform to integrate microbiome analysis to dissect host microbiome interactions, epigenetics changes, and microbiome trajectories in health and early life.

**Figure 4:**
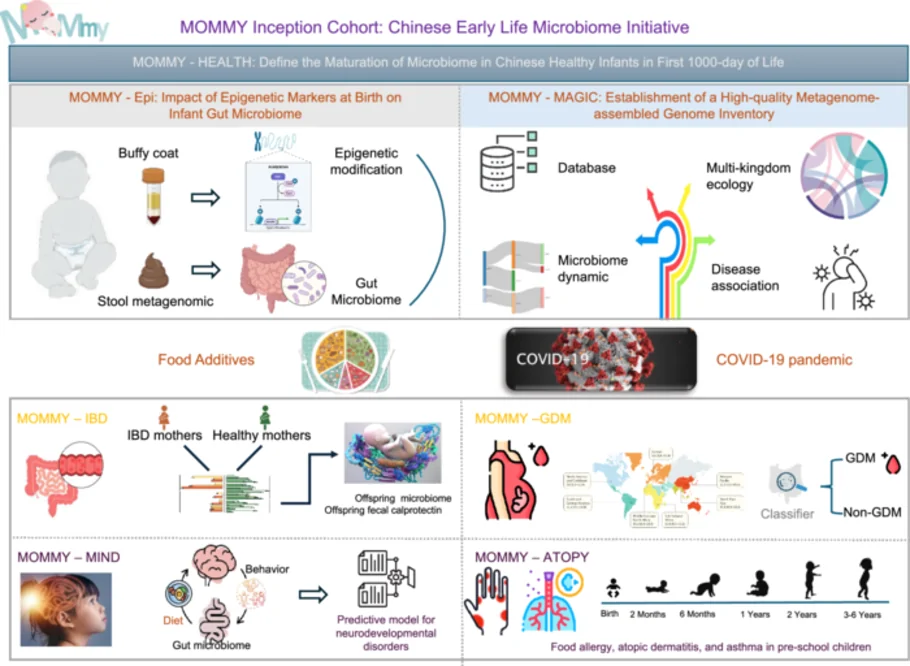
MOMMY inception cohort projects. Studies derived from the MOMMY: MOMMY‐HEALTH, MOMMY‐Epi, MOMMY‐MAGIC, MOMMY‐FA, MOMMY‐COVID, MOMMY‐IBD, MOMMY‐GDM, MOMMY‐MIND, and MOMMY‐ATOPY.

To maximize the scientific yield, we organized the MOMMY inception cohort into two layers of projects (Figure 4)**:** ***(1) Lay the basic infrastructure (i–v):*** to define the healthy early life microbiome, develop early life microbiome reference database, multi‐omics data set, state‐of‐the‐art computational and statistical tools, environmental exposure (COVID pandemic and nutrition/dietary) assessment; ***(2) Expand to clinically‐oriented microbiome‐associated conditions (vi–x):*** Maternal complications: Gestational diabetes mellitus (GDM) and inflammatory bowel disease (IBD); Offspring diseases: pediatric allergic disease and NDD. These studies will follow the dynamics of these conditions through multi‐omic analyses of multiple measurement types over time, including changes in microbial community composition, viromes, metabolomic profiles, gene expression and protein profiles from both host and microbiome, and host‐specific properties such as genetic, epigenomic, along with other study‐specific features. All sequences and multi‐omic data, clinical information, and tools from the MOMMY inception cohort will be housed in the MAGIC Data Coordination Center in Hong Kong.

(i) ***MOMMY ‐ HEALTH: Machine Learning Model to Define the Maturation of Microbiome in Chinese Healthy Infants in the First 1000‐day of Life*** In the first 1000‐day of life, the microbiome of infants goes through significant changes that have long‐term implications for health and disease [55, 56]. Understanding the development of the microbiome in healthy infants is crucial, as it can help identify potential biomarkers, elucidate the dynamics of microbial colonization, and inform the development of interventions to promote optimal microbial growth. Healthy infants are defined based on gestation weeks (full‐term), physical growth (growth *z*‐score between −2 and +2), and the absence of any auto‐immune diseases diagnosed during the specific study periods. In this MOMMY ‐ HEALTH, we will establish a standard reference of the composition of the microbiome throughout the first 1000‐day of life, using metagenomic data generated from the MOMMY inception cohort. Moreover, we will develop a machine learning model to estimate the age of infants based on their microbiome developmental trajectory. Through it, we will identify key microbial community dynamics and patterns associated with microbiome maturation. Any deviation of the microbiome age may indicate potential developmental delays, requiring early intervention such as personalized nutrition and health recommendations. Besides, insights into the impact of key factors in early life, such as breastfeeding, weaning, solid food introduction, and environmental exposures on microbiome maturation are expected. The microbiome‐based machine learning model will lead to a better understanding of the microbiome and its relationship to age and development, which can facilitate the development of new approaches for promoting healthy growth and development in early life.
(ii) ***MOMMY ‐ Epi: Impact of Epigenetic Markers at Birth on Infant Gut Microbiome*** Interactions between host genetics and gut microbiota reveals microbial signatures that are common and characteristic of complex diseases [57, 58]. However, most current research on the genetic‐microbiome interaction focuses on adults. The gut microbiome of adults is affected by factors such as age, geographical environment, diet, disease, etc. Limited interaction between early life gut microbiome and host genetics was identified in current large‐scale studies. DNA methylation is the most extensively studied epigenetic mechanism, first appearing during cellular differentiation in embryogenesis. A cohort study in the US explored the connections between prenatal exposure, epigenetic alterations, and the development of the microbiome [59]. This study identified a set of CpG sites with modified methylation patterns in the cord blood of infants born to mothers with asthma. These sites were enriched with elements responsive to microbes and were associated with both microbial colonization in the upper respiratory tract at 2 years and the onset of childhood asthma. However, it remains unclear how epigenomic configurations at birth may influence the impact of perinatal factors on the initial establishment and growth of the infant gut microbiome.
  We are building up a comprehensive integrative early life microbiome platform with the aim to understand the complex host–environment–microbe interactions in microbiome seeding and development in early life. We will focus on the epigenome at birth, a factor that is poorly studied, which controls host gene expression and may partially program gut microbiota development and health outcomes. In MOMMY‐Epi, with an identified core subset of 968 families from the MOMMY inception cohort, we will perform a comprehensive integrative analysis metagenomic sequencing of the maternal fecal samples collected at the third trimester and infant fecal samples collected at birth and 2 months of age, as well 16S and ITS amplicon sequencing for breastmilk samples and vaginal samples. Beyond metagenomics, we will also utilize other omics, including epigenomes of cord blood samples derived from whole genome bisulfite sequencing to provide insight into host factors in this scenario. This project will conduct bioinformatics big data integration on the whole genome/epigenome sequence and intestinal microbiome to characterize how the early life microbiome seeding processes are affected by host genomes and pinpoint the keystone microbiome taxa that lead to healthy gut homeostasis.
  Integrative analysis of the epigenome, exposome and microbiome in early life will advance our knowledge of the impact of environmental exposures on the interplay between host epigenome and microbiome in early life and associated disease outcomes, hence facilitating the development of integrated omics markers for more precise and sensitive early disease detection. This project will also generate the first single databases that contain comprehensive data on both microbiome from multiple body sites and epigenomes. Such a database will drive and accelerate the new direction of microbiome research and development.
(iii) ***MOMMY ‐ MAGIC: Establishment of a High‐quality Metagenome‐assembled Genome Inventory*** During the establishment of the cohort and subsequent analyses on microbiome data in early life, we identified a research gap that early‐life microbiota data are underrepresented in existing databases, hampering accurate profiling of the infant gut microbiome. To address this gap, we generated a Metagenome‐Assembled Genome Inventory for Children (MAGIC) from more than 19,559 early‐life (0–7 years) publicly available early‐life metagenomes in combination with 613 metagenomes generated from our MOMMY inception cohort [60]. In this project, we generated an early‐life gut virome database that will enable complete and accurate profiling of infant/child gut virome. We utilized state‐of‐the‐art bioinformatic tools to generate the MAG inventory for early life. The MAGIC data entries and annotations are available at https://github.com/ohmeta/MAGIC and https://zenodo.org/doi/10.5281/zenodo.10369093. The code for the generation of prokaryotic MAGs and viral MAGs from raw sequencing reads is available at https://github.com/ohmeta/metapi. A tutorial for using MAGIC in the Phanta pipeline is available at https://github.com/ohmeta/MAGIC. Study details have been published in the reputable journal *Cell Host Microbe* [60]. In the next phase, we aim to double the number by adding samples from MOMMY as well as newly available public datasets.
  The MAGIC database^43^, therefore, not only recovers previously uncharted genomic information but also enables comprehensive characterization of the dynamics of early‐life microbiomes, identification of keystone species, and strain‐level study of target species. This database will be complementary to our prospective mother‐baby cohort, which we have collected over 58,000 biological samples (stool, serum, urine, vagina swab, buccal swab) and 25,000 web‐based clinical metadata based on validated questionnaires from parents and their infants at multiple time points in the pre‐, peri‐ and postnatal period. This exemplifies that our comprehensive databases can provide significant benefits to the industry, particularly startups and companies involved in diagnostic biomarker development for early‐life.
(iv) ***MOMMY ‐ FA: The Impact of Food Additives on Maternal and Child Health Outcome*** Accumulating evidence suggests that food additives can lead to altered gut microbiome composition [25, 26]. Yet the impact of food additive intake on early life gut microbiome and prenatal health has not been explored. We have developed and validated a food frequency questionnaire used to estimate additive intake over the preceding 12 months for the parents, which is collected at baseline, postnatal months 6 and 18. These questionnaires were developed to suit specific study regions by taking into consideration the dietary habits of the population living in that region. In brief, the questionnaire addresses intake frequency and amount of 26 food lists over the past 12 months. The food lists were created based on “Codex General Standard for Food Additives” food sub‐categories that contain additives of interest. Maximal exposure to nine food additives, namely three emulsifiers (CMC, carrageenan [CRN], and P80), three sweeteners (aspartame [ASP], saccharin [SAC], and sucralose [SUC]), and three humectants, preservatives, or coloring additives (aluminosilicate [AlSiO], sulfites [X(SO_2_)], and titanium dioxide [TiO_2_]) were assessed and estimated in mg/year based on the regional maximal permissible level (MPL) of additives in each food list, or where MPL does not exist, based on concentration data from the literature. We also established a mass‐spectrum (MS) based food additive levels measurement in different biological samples, including urine, serum, and stool.
  In a subgroup of pregnant women recruited to the MOMMY inception cohort, we will conduct a separate investigation into the potential impact of food additives. In MOMMY‐FA, the impact of food additives intake on two common perinatal diseases, GDM and offspring large for gestational age (LGA), and gut microbiome composition at late pregnancy will be assessed. Maternal stool collected at MG3 and infants' stool samples from birth till 1‐year‐old will be used to assess the potential transmission variance among different food additive intake groups. In the MOMMY cohort, using semi‐quantitative questionnaires (parental current and past food additive intake) and mass‐spectrum (MS) based food additive levels measurement in different biological samples, we are able to unravel the cross‐generational health impairment of food additives and whether it involves reforming the parental and postnatal gut microbiome.
  By observing the contribution of food additives on perinatal health and gut microbiome composition, we can develop more effective recommendations to promote healthy growth and development in infants and young children.
(v) ***MOMMY ‐ COVID: Impacts of COVID‐19 Pandemic on Early Life Gut Microbiome***
  The development origins of health and disease (DOHaD) hypothesis suggests that adverse exposure during early life, particularly in utero, may substantially influence later‐life health and disease status [61]. Analogous to DOHaD, the early life, especially the first 1000 days (from conception to 3 years of age) of life, is critical for gut microbiota establishment and development. The above evidence leads us to propose the hypothesis that the gut microbiome may be a key component contributing to the DOHaD concept [62]. Notably, MOMMY was established in Sep 2019 and “perfectly” experienced the COVID‐19 pandemic from 2020 to 2023. The COVID pandemic, as a natural experiment, provides a unique opportunity to examine the impact of early‐life adverse exposure on the adult and later‐life gut microbiota and the role of gut microbiota in the DOHaD. MOMMY‐COVID provided the unique opportunity to assess the effect of increased hygiene standards on early gut microbiome maturation. These insights will underscore the importance of considering the microbiome when evaluating hygiene measures and the need for future research to ascertain the role of the gut microbiome in disease development. Study details have been published in the reputable journal *Gut Microbes* [63].
(vi) ***MOMMY ‐ GDM: Early Life Microbial Determinants in Gestational Diabetes Mellitus (GDM)*** GDM, one of the most common gestational complications, increases the risk of long‐term complications in the mother, especially obesity, impaired glucose metabolism, and cardiovascular disease [64]. GDM can increase the risk of infant complications as well, such as neurodevelopment disorders and obesity [65, 66]. In MOMMY‐GDM, in this study, we investigated the gut microbiome profile of mothers during pregnancy and infants during the first year of life from a large‐scale longitudinal cohort of 264 mother‐baby dyads with 1566 total metagenomic sequence stool data in Hong Kong. We identified several maternal bacteria features associated with GDM status and glycemia levels during whole gestational periods. To our knowledge, this pioneering study identified that GDM is deeply fingerprinted on the gut microbiome of male offspring up to 12 months of life. Male infants born to GDM mothers have different gut microbiome, which associates with larger head circumference growth. Our new findings suggested the maternal prenatal gut microbiome was relevant to the children's neurodevelopment in the first year of life. However, the impact of GDM on offspring neurodevelopmental sex differences should be considered not only for brain damage but also for brain protection. Future research is necessary to understand the long‐term consequence of over‐growth in these infants and whether microbiota modulation serves as a potential approach to mitigate and prevent these risks. Study details have been published in the reputable journal *Cell Host & Microbe* [67].
(vii) ***MOMMY ‐ IBD: The Impact of Food Additives on Gut Microbiota and Inflammatory Bowel Disease*** IBD, including conditions like Crohn's disease (CD) and ulcerative colitis (UC), is characterized by chronic inflammation of the gastrointestinal (GI) tract, and its incidence and prevalence are increasing globally [68]. Factors such as maternal smoking, diet, antibiotics, and breastfeeding during early life can impact the risk of IBD through the gut microbiome [69, 70]. However, there is a dearth of prospective human studies investigating the relationship between maternal diet, microbiome, and IBD. The consumption of ultra‐processed foods and food additives has become increasingly common in modern diets [23]. Despite preclinical evidence, no human studies have yet examined the impact of maternal intake of food additives in mothers with IBD on the microbiome of their infants [71]. This multi‐center cohort study therefore provides an unprecedented opportunity to test the hypothesis that maternal food additive intake impacts the maternal gut microbiome, potentially affecting themicrobiome/inflammation status of their offspring up to 6–7‐year‐old. In MOMMY‐IBD, in the short term, we will focus on exploring the impact of diet on gut microbiota and subsequent host immune dysfunction that underlies mechanisms of gut inflammation in early life [72, 73]. Our long‐term goal is to modulate the intestinal microbiome with novel human‐derived bacteria consortia and dietary interventions to inhibit maternal inflammatory and immune pathways for the prevention of IBD. We will perform multi‐level immune profiling on infant stool and blood samples to capture both mucosal (stool samples) and systemic (blood samples at 4‐year‐old postnatal) immune responses. From stool samples, we will measure a panel of immune mediators using multiplex immunoassays. This will include key cytokines, such as pro‐inflammatory markers and immunoglobulin A (IgA), fecal calprotectin and lipocalin‐2 (LCN2) [74, 75, 76]. From blood samples, plasma cytokine and immune cell phenotyping (flow cytometry) will be performed by peripheral blood mononuclear cells (PBMCs) to characterize key cell populations (e.g., T‐regulatory cells (Tregs), Th1, Th2, Th17 cells, B cells, and innate immune cells). Three core pillars of our research directions: (A) Deciphering crosstalk and mechanisms of diet‐intestinal microbiota in the immunopathogenesis of IBD in early life; (B) Dissecting diet additives' impact on host early immunity development in infants born to IBD and healthy mothers; (C) Modulation of intestinal microbiota with novel bacteria consortia‐secreting immunomodulatory metabolites that target inflammation.
(viii) ***MOMMY ‐ ATOPY: Childhood Allergy and the microbiome – precision health for life*** Eczema causes substantial physical and psychosocial morbidities in these victims, and the current mainstay treatments with corticosteroids and other anti‐inflammatory drugs are associated with local and systemic adverse effects [77]. The early‐life gut microbiome is associated with the development of allergic diseases in children, including eczema, wheezing (asthma), and allergic sensitization. Among these allergic diseases, eczema affects up to 20% of children and represents one of the earliest manifestations of the atopic march [78, 79]. Therefore, it is important to identify the factors affecting eczema development, especially in Chinese children, who showed the highest incidence rate of eczema in the first year of life among other ethnic groups.
  In this MOMMY ‐ ATOPY, the incidence of eczema within 1‐year‐old (up to July 2023) is around ≈13% (156/1200 subjects). Leveraging the ongoing follow‐up of the MOMMY inception cohort, these estimations highlight the potential of MOMMY‐ATOPY for future prospective studies elucidating the role of the microbiome in pediatric allergic disease. Taking advantage of the allergy‐related clinical data collected systematically and prospectively, we will conduct a longitudinal nested case‐control study of atopic diseases across the first 6 years of life. Estimation of sample size: The relative microbiota maturity in our previous studies was used for sample size calculation. Relative microbiota maturity was defined as the predicted microbiota age of the infant minus the microbiota age of healthy infants at the same chronological age, as determined by the smoothing spline. Based on our pilot data, we assume an absolute difference of 5.1 (with SD of 18.6) for relative microbiota maturity between infants with or without eczema. A sample size of 220 in each group can detect this effect size with 80% power and two‐sided 5% type I error for a two‐sample *t*‐test. According to the accumulative 20% prevalence of atopic diseases, 1100 consecutive infants will be recruited and assessed from the MOMMY inception cohort.
  Our specific objectives were to (i) characterize the development of the gut, skin, and nasopharyngeal microbiome in a Chinese birth cohort during the first 6 years of life; (ii) identify risk factors for atopic disease development from maternal, perinatal, fetal, and environmental variables; and (iii) identify gut, skin, and nasopharyngeal microbiome signatures for prevention and treatment of food allergy, atopic dermatitis, and asthma in pre‐school children. We will perform shotgun metagenomic sequencing for the fecal microbiome profile and 16S rRNA amplicon sequencing for the skin and nasopharyngeal microbiome profile. We expect to unveil the role of interaction between environmental factors and gut, skin, and nasopharyngeal microbiome in atopic diseases and identify gut, skin, and nasopharyngeal microbiome signatures for the management of food allergy, atopic dermatitis, and asthma in preschool children.
(ix) ***MOMMY ‐ MIND: Identification of Fecal Microbiome Biomarkers to Predict Risk of Neurodevelopmental Disorders*** The prevalence of NDDs, such as ASD or attention deficit hyperactivity disorder (ADHD), has increased over recent years [80, 81]. Emerging studies suggest the actual involvement of the gut microbiome in regulating brain development [82, 83]. Nevertheless, any disturbance that could negatively influence brain functionality could also possibly lead to a range of neurodevelopment disorders [84]. Critical developmental windows that influence early behavioral outcomes have been identified, including both the prenatal environment and early postnatal colonization periods. The clearest data regarding the role of gut microbiota on neurodevelopment and psychiatric disorders are from animal studies; however, human data have begun to emerge, including an association between early colonization patterns and cognition [85].

In this MOMMY‐MIND study, we aim to leverage a well‐characterized prospective inception cohort MOMMY to determine the role of fecal microbiome markers as well as dietary factors in infants for predicting and detecting risk of NDDs in children, especially children with ASD, ADHD, and anxiety disorders. Estimation of sample size: The predicted probabilities for NDDs in our previous studies [4, 86] were used for sample size calculation. The mean difference of predicted probabilities between subjects with and without NDDs was 0.12 (0.35 vs. 0.47). The population standard deviation was 0.138 based on our over 1600 Hong Kong population data set. Based on these preliminary data, 250 participants per group are required for a two‐tailed chi‐square test with a type I error of 0.05 and a power of 95%. According to the accumulative 25% prevalence of NDDs, 1000 consecutive infants will be recruited and assessed. A sample of healthy children, matched on gender, and sex, would serve as controls. All children will also be profiled by intelligence quotient (IQ), socio‐demographics, and family history of psychiatric conditions. We hypothesize that early life microbiome signatures are associated with, and may be predictive of the risk of NDDs in infants. We aim (1) to identify gut microbial markers in the first year of life, associated with, and predictive of ASD in preschool children; (2) To determine early life gut microbial signatures associated with, and predictive of ADHD and anxiety disorders in preschool children; (3) To delineate the association of early life gut microbiome and IQ in pre‐school children; (4) To investigate the impact of maternal diet and infants' diet on microbiome and NDDs in pre‐school children. The clinical identification of neurodevelopmental/psychiatric disorders will be a 2‐stage process, first by parent‐filled questionnaire, followed by a standardized confirmatory diagnostic interview.

## 4 POTENTIAL BIAS AND LIMITATIONS

There are valuable contributions of established birth cohorts, such as CHILD (Canada), GUSTO, and S‐PRESTO (Singapore), in advancing early‐life microbiome research. Key findings from these cohorts, including environmental and maternal determinants that influence the development of the infant's multi‐kingdom microbiome (bacterial and fungal), as well as links to child health outcomes (growth, atopic eczema) [87, 88, 89, 90], have been foundational to the field. Most large‐scale microbiome cohorts are Western (e.g., CHILD, TEDDY) or city‐specific (e.g., GUSTO). MOMMY's inclusion of diverse Chinese populations with distinct dietary, environmental, and genetic influences on microbiome development addresses the underrepresentation of these groups in microbiome research. In the meantime, it may introduce potential biases in our cohort and may limit the generalizability of our findings. Therefore, our results will also be validated in other birth cohorts using a publicly available data set. Unlike many cohorts with limited follow‐up, MOMMY tracks mother‐infant dyads from pregnancy through early childhood, capturing dynamic microbiome transitions (e.g., weaning, antibiotic exposures) with high‐resolution sampling. MOMMY combines microbiome data with detailed metadata (e.g., maternal diet, urban/rural disparities) and host genomics, enabling mechanistic insights into culturally specific drivers of microbiome assembly.

## 5 CONCLUDING REMARKS

In this perspective, we provide a profile of the bio‐samples and comprehensive assessments collected in MOMMY and define the MOMMY inception cohort used to evaluate microbiome determinants of major clinical outcomes. The long‐term goal of MOMMY is to identify environmental, dietary, and microbial determinants (including psychosocial factors) in early life that trigger (perhaps through genetic susceptibility) or protect against common childhood diseases, allergic diseases, (eczema, wheezing, asthma, and allergic sensitization) and NDD. Operating multi‐omics data from various biosamples, the MOMMY cohort will significantly enhance the resolution of mechanisms of microbiome transmission from mother to baby. Taken together, the MOMMY cohort provides the translational value of innovations for disease early diagnosis, prevention, or treatment based on strain‐level manipulation of the gut microbiome.

Overall, that's just the start of the effort and will be the first largest prospective Asia population‐based birth cohort study that is ambitious to uncover the link between the parental and newborn microbiome, segregating favorable and unfavorable taxa with multiple measures of both dietary intake on health and disease in early life development. As a resource, these results will aid both in the utilization of the gut microbiome as a biomarker for disease risk and in strategies for reshaping the microbiome to improve personalized health, which will have significant implications for preventive interventions and guide future public health and government policy development.

## AUTHOR CONTRIBUTIONS

**Lin Zhang**: Data curation; writing—original draft; writing—review and editing; formal analysis; visualization. **Yingzhi Liu**: Data curation; writing—original draft; writing—review and editing; visualization; formal analysis. **Shilan Wang**: Data curation; writing—original draft; writing—review and editing; visualization; formal analysis. **Jessica Yuet‐Ling Ching**: Data curation; writing—review and editing; formal analysis. **Wing Hung Tam**: Methodology. **Ting Fan Leung**: Methodology. **Tak Yeung Leung**: Methodology. **Paul K. S. Chan**: Methodology. **Joyce W. Y. Mak**: Methodology. **Chun Pan Cheung**: Methodology. **Hein Min Tun**: Methodology. **Eugene B. Chang**: Writing—review and editing. **Orlando DeLeon**: Writing—review and editing. **Qitao Huang**: Methodology. **Xiaoqian Chen**: Methodology. **Huiyi Huo**: Methodology. **Yinglei Miao**: Methodology; Funding acquisition. **Pui Kuan Cheong**: Methodology. **Ka Long Ip**: Methodology. **Yuk Ling Yeung**: Methodology. **Mei Kam Chang**: Methodology. **Chunmei Lyu**: Methodology. **Hongju Yang**: Methodology. **Bona Li**: Methodology. **Yushuo Fan**: Methodology. **Yang Sun**: Methodology; resources; funding acquisition. **Suhua Jiang**: Methodology; resources; funding acquisition. **Siew Chien Ng**: Conceptualization; funding acquisition; writing—review and editing; resources; methodology. **Francis Ka Leung Chan**: Conceptualization; methodology; writing—review and editing; funding acquisition; resources. All authors have read the final manuscript and approved it for publication.

## CONFLICT OF INTEREST STATEMENT

F.K.L.C. serves as the Principal Investigator for the Fecal Microbiota Transplantation Service under the Hospital Authority (HA). He is a Board Director of EHealth Plus Digital Technology Ltd, an HA‐owned subsidiary driving the eHealth+ program to transform the Electronic Health Record Sharing System into a comprehensive digital healthcare platform and advance other IT initiatives within the eHealth ecosystem. F.K.L.C. is a shareholder of GenieBiome Holdings Limited and the cofounder, nonexecutive Board Chairman, and nonexecutive Scientific Advisor of its wholly owned subsidiary, G‐NiiB GenieBiome Limited. Similarly, he is a shareholder of MicroSigX Diagnostic Holding Limited and the cofounder, nonexecutive Board Chairman, and nonexecutive Scientific Advisor of its wholly owned subsidiary, MicroSigX Biotech Diagnostic Limited. He also serves as a Director of the Hong Kong Investment Corporation Limited and a member of the Steering Committee for the RAISe+ Scheme under the Innovation and Technology Commission. Furthermore, he is the Codirector of the Microbiota I‐Center (MagIC) Ltd. F.K.L.C. receives advisory fees and speaker honoraria from AstraZeneca and Comvita New Zealand Limited, as well as patent royalties through affiliated institutions for microbiome‐related applications. S.C.N. has served as an advisory board member for Pfizer, Ferring, Janssen and Abbvie and received honoraria as a speaker for Ferring, Tillotts, Menarini, Janssen, Abbvie and Takeda; has received research grants through her affiliated institutions from Olympus, Ferring and Abbvie; is a founder member of GenieBiome Ltd; is a shareholder of GenieBiome Holdings Limited; GenieBiome Limited is wholly owned by GenieBiome Holdings Limited; is a nonexecutive Board director and nonexecutive scientific advisor of GenieBiome Ltd and its holding company which is non‐remunerative; is a founder member of MicroSigX Biotech Diagnostic Limited; is a shareholder of MicroSigX Diagnostic Holdings Limited; GenieBiome Limited is wholly owned by GenieBiome Holdings Limited; is a nonexecutive Board director and nonexecutive scientific advisor of MicroSigX Biotech Diagnostic Limited and its holding company which is non‐remunerative; and receives patent royalties through her affiliated institutions. F.K.L.C., S.C.N., L.Z., and H.M.T. are named inventors of patent applications held by the CUHK and MagIC that cover the therapeutic and diagnostic use of microbiome. The remaining authors declare no conflicts of interest.

## ETHICS STATEMENT

This study has been registered at ClinicalTrials.gov (NCT04117321) and has been approved by the Joint Chinese University of Hong Kong – New Territories East Cluster Clinical Research Committee (reference number 2019.243).

## Data Availability

The MOMMY Study welcomes collaboration, and we are keen to establish MOMMY as a research platform. To protect participant's privacy and comply with the informed consent, participant metadata used in the current study cannot be deposited in public repositories. According to our institutional data sharing policy, data sharing requests may be submitted with a written proposal as a part of a collaboration effort. The proposal should detail the intended use of the data. These requests are reviewed based on scientific merit and ethical considerations, including participant consent. Data sharing will be undertaken if the proposed projects have a sound scientific rationale or potential societal benefit. We ensure that restrictions related to subject confidentiality and consent are addressed by aggregating and anonymizing identifiable data. Additionally, all indirect identifiers that could lead to deductive disclosures will be removed. Data recipients are required to enter formal data sharing and collaboration agreements, which describe the conditions for release and requirements for data transfer, storage, archiving, publication, and intellectual property. Supplementary materials (graphical abstract, slides, videos, Chinese translated version, and update materials) may be found in the online DOI or iMeta Science http://www.imeta.science/imetaomics/.
